# The efficacy of a novel porcine-derived collagen membrane on guided bone regeneration: a comparative study in canine model

**DOI:** 10.1186/s12903-025-05930-6

**Published:** 2025-05-29

**Authors:** Anh Thi Mai Nguyen, Euphemie Landao-Bassonga, Elias D. Kontogiorgos, Clair Lee, Tak Cheng, Hien Chi Ngo, Brent Allan, Mithran Goonewardene, Lynne A. Opperman, Minghao Zheng

**Affiliations:** 1 Centre for Orthopaedic Research, Medical School, The University of Western Australia, Nedlands, WA Australia; 2 Perron Institute for Neurological and Translational Science, Nedlands, WA Australia; 3 Texas A&M College of Dentistry, Dallas, TX USA; 4 Dental School, The University of Western Australia, Nedlands, WA Australia

**Keywords:** Guided bone regeneration, Collagen membrane, Animal study, Dental implant, Canine

## Abstract

**Background:**

This study aimed to evaluate the performance of the novel Striate + collagen membrane in a canine model of guided bone and tissue regeneration (GBR) with dental implant placement.

**Methods:**

Eighteen mature beagle dogs were used in this split-mouth study. After having their premolar teeth extracted, GBR with immediate implant placement was performed on all study subjects. study subjects. The study treatments were: control group (implant + no membrane); BG-group (implant + Bio-Gide membrane); and SG-group (implant + Striate + membrane). Six dogs were sacrificed at 4-, 8- and, 12-weeks post-treatment for radiographic (µCT) assessment, histological examination and histomorphometric analysis.

**Results:**

µCT assessment showed that all groups exhibited increased bone formation from 4-weeks to 12-weeks post-treatment. There was no statistically significant difference in mean BV/TV between all 3 groups at weeks 4 and 8. At week 12, BV/TV was significantly higher in SG and BG-groups compared to control group. Assessment of bone microarchitectural parameters showed that animals in SG-group exhibited significantly higher Tb.N, O.Wi and lower Tb.Sp, suggesting more favorable mature bone structure. A significant increase in the number of osteoblasts on bone surface was also seen in SG-group. Histological assessment showed that SG-group displayed early signs of bone-to-implant contact at 8 weeks. While control sites showed early ingrowth of epithelium and connective tissue into the defects, infiltration of inflammatory cells, incomplete bone formation and limited bone to implant contact, use of a barrier membrane resulted in significant bone infill, mature bone formation with good bone to implant contact. and limited soft tissue invasion.

**Conclusion:**

This study demonstrated superiority of Striate + collagen membrane to promote good bone formation and prevent unwanted epithelial infiltration in a canine mode of GBR.

**Supplementary Information:**

The online version contains supplementary material available at 10.1186/s12903-025-05930-6.

## Background

Guided bone regeneration (GBR) is commonly used in dentistry, and it involves the use of bone substitutes and a barrier membrane [1–3]. Bone substitute materials are used to bulk-fill the defects, while the barrier membrane is utilized to prevent the ingrowth of faster-growing epithelial soft tissues during healing [1]. GBR has a high success rate for correcting dehiscence and fenestration defects, and reconstructing bone volume associated with implant placement [4, 5]. In a systematic review of 238 patients with 374 implants placed using GBR, the overall implant survival rate of 95.7% was reported, regardless of barrier membrane and grafting material type [4]. The key biological characteristics of a barrier membrane include inhibiting soft tissue invasion, promoting bone regeneration, enhancing vascularization, and biocompatibility with surrounding tissues [6, 7].

Multiple synthetic membranes have been studied for application in GBR. These include resorbable and non-resorbable membranes. The non-resorbable membranes show great biocompatibility and ability to promote bone regeneration [8], however, a highly desirable property is bioresorbability, as this would eliminate the need for a second surgery to remove the membranes. Thus, complete resorption after bone remodeling is an important parameter when selecting the most ideal barrier membrane for GBR [6, 7, 9]. Several biodegradable materials have attracted the attention of dental researchers. Rider et al. assessed the usage of a novel bioabsorbable pure magnesium membrane [10], whereas Reis et al. have examined a rigid hydroxyapatite resorbable membrane for GBR applications [11]. However, complications during the healing process due to tissue inflammatory responses, unsatisfactory bone regeneration outcomes, and membrane exposure were reported in both cases.

Collagen is a natural protein that makes up the framework of tissues [12, 13]. Its crucial roles in multiple cellular processes and outstanding biocompatibility are key features for its widespread use and multidisciplinary applications [7, 14, 15]. Collagen-based biomaterials have been demonstrated to regulate and promote tissue regeneration [16]. In GBR, collagen membranes are the most used resorbable barrier membrane [12, 17]. Strong evidence shows that the combined application of bone graft materials and collagen membranes during implant placement leads to more successful rehabilitation of bone defects surrounding the implants [18, 19].

Several studies suggested that the degree of crosslinking of collagen fibers prolongs resorption and maintains a favorable microenvironment for bone regeneration [20, 21]. However, crosslinking has been shown to elicit more adverse events leading to insufficient bone regeneration as compared to native collagen membranes [22, 23]. A meta-analysis of membranes used in GBR has shown that crosslinked membrane exposure rates were around 30% higher than that of non-crosslinked membranes and correlated with compromised GBR outcomes [24]. In comparison, GBR performed with non-crosslinked membranes exhibited significantly greater tissue integration [21] and induced earlier angiogenic patterns [25]. Therefore, on the balance of the available evidence, natural non-crosslinked collagen membranes remain the better option for use as barrier membranes for GBR applications.

Here we have developed a resorbable, natural non-crosslinked collagen membrane of porcine origin. In previous studies, we showed that use of the collagen membrane results in repair of cortical bone defects in rat [26, 27] and rabbit [9] models by upregulation of pro-osteogenic factors, inducing cellular recruitment at the implant site, enhancing tissue vascularization and promoting bridging of gaps in the cortical bone defect [9, 26]. The objective of this study was to evaluate the use of resorbable, natural non-crosslinked collagen membrane, marketed as Striate +, for GBR in a canine model. We selected the canine model as the periodontal tissues in dogs are more like humans and the larger jaws and dentition would present easier access during the operation [28]. We hypothesized that as a GBR barrier membrane, Striate + is effective in the restoration of cortical bone defects in a canine model. Assessments included radiographic (uCT) and histomorphometric analyses to evaluate bone regeneration, biocompatability and barrier membrane function following simultaneous dental implant placement.

## Materials & methods

### Biomaterials

Striate +, implantable collagen membrane is manufactured by Orthocell, Australia. The maximum size of the membrane is 30 × 40 mm and contains cell-free native type I collagen with porcine origin. The patent was developed at the University of Western Australia and licensed by Orthocell in Australia [9]. Geistlich Bio-Gide resorbable bilayer membrane (Geistlich Pharma; Switzerland) was used as the positive comparator control. Bio-Gide is a porcine-derived biocompatible and resorbable bilayer collagen membrane containing type I and III collagen used as a barrier membrane for GBR to promote tissue healing and bone remodeling [29, 30]. Striate + and Bio-Gide exhibit similar physicochemical and biological characteristics [12]. Other items used in this study include Geistlich Bio-Oss spongious bone substitute (Geistlich Pharma; Switzerland) and Xive S plus implant (3.8 × 9.5 mm^2^ (L9.5), and 3.8 × 8 mm^2^ (L8); Dentsply Sirona; NC, USA).

### Study design

**Fig. 1:**
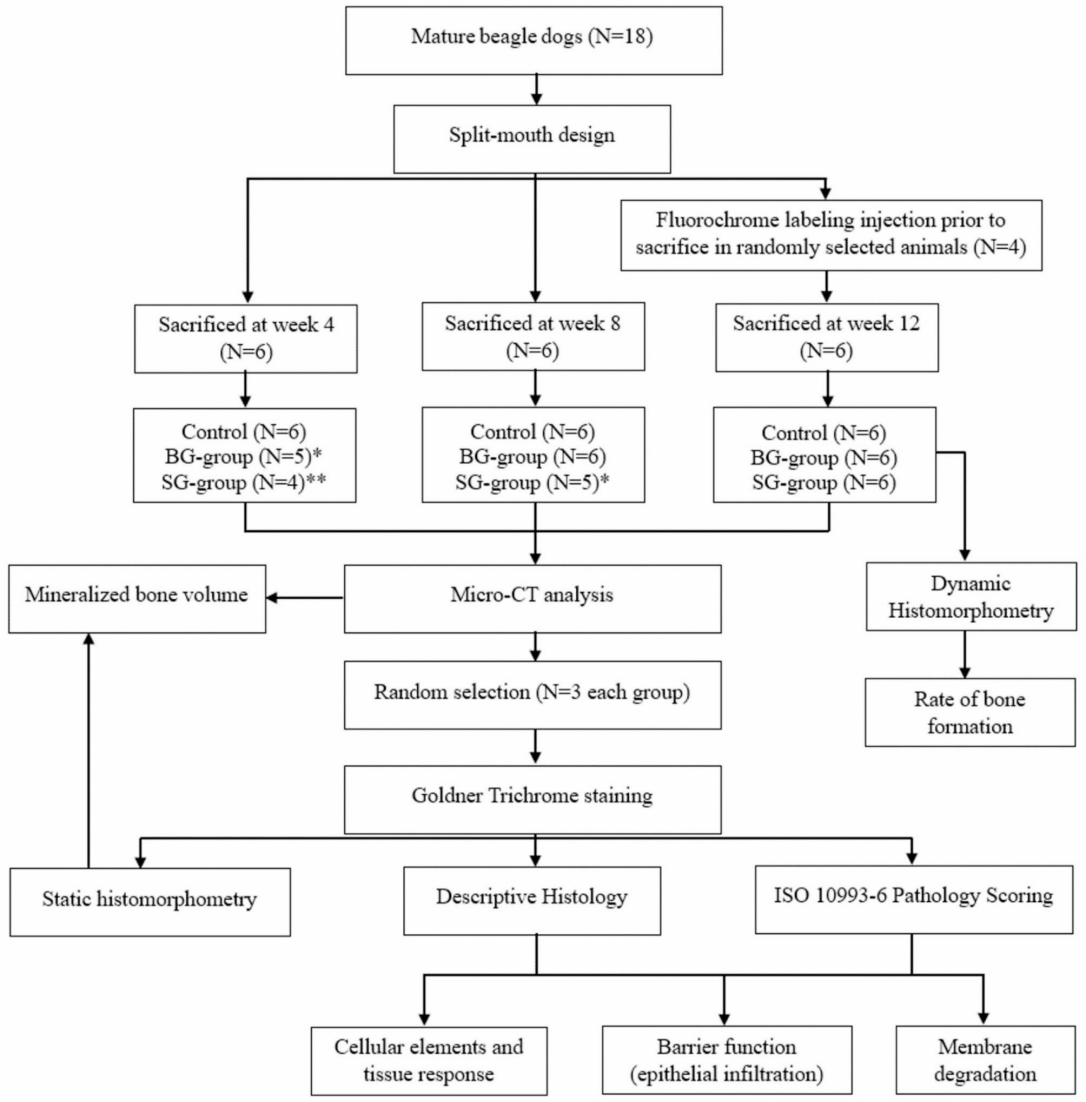
Study workflow. * one sample was excluded from the analysis due to loss of implants; ** two samples were excluded from the analysis due to loss of implants

A split-mouth design was used, with dental implants placed immediately in premolar regions after extraction. Sockets were filled with bone grafting material and covered by a collagen barrier membrane. Animals were assigned to one of 3 study groups (Fig. 1): control group (implant and bone grafting material with no membrane); implant and bone grafting material with Bio-Gide barrier membrane (BG-group); and implant and bone grafting material with Striate + barrier membrane (SG-group). Study endpoints were 4-, 8-, and 12-week post-surgery.

### Animals

Eighteen mature beagle dogs (from Marshall BioResources; NY, USA) were used in this canine model of GBR. They were both male and female, approximately 2 years old and weighing 8 to 12 kg. Animals were maintained in accordance with the Texas A&M College of Dentristry animal husbandry SOPs and under approval from the Texas A&M School of Dentistry Institutional Animal Care and Use Committee (IACUC approval 2018-0090-CD). All study protocols followed the ARRIVE (Animal Research: Reporting of In Vivo Experiments) guidelines.

### Surgical procedures for tooth extraction and implant placement

Animals were anaesthetized with intramuscular injection of 1.1–2.2 mg/kg Ketamine (Animal Health; TX, USA) and 0.11 mg/kg Xylazine (Animal Health; TX, USA). Bilateral mandibular blocks were performed using 2% Lidocaine hydrochloride with 1:100,000 Epinephrine (Novocol Pharmaceutical; Canada). All teeth were cleaned prior to extraction and insertion of implants. Each selected premolar (107, 207, 306, 308, 406 or 408 sites) was sectioned to facilitate atraumatic extraction. Following tooth extraction, the implant site was prepared according to the implant manufacturer’s instructions with a 3.8 mm diameter Twist Drill Crestal (Dentsply Sirona; NC, USA). A titanium threaded implant (Xive S Plus; Dentsply Sirona; NC, USA) was then placed in the tooth socket and the remaining void was filled with Bio-Oss spongious bone substitute (Geistlich Pharma; Switzerland). A collagen barrier membrane, either Bio-Gide (Geistlich Pharma; Switzerland) or Striate+ (Orthocell; Australia), was placed over the defect and tucked under the gingiva, sealing the socket. The membranes were trimmed to size and placed smooth side up over the implant site, extending 2–3 mm beyond the GBR margin. The gingiva was then closed with interrupted sutures (Monocryl 4 − 0; Ethicon; NJ, USA) to cover the membrane. Control sites were treated identically except no membrane was placed (Supplementary Fig. 1).

Animals were closely monitored until recovery from anaesthesia and then returned to the animal facility. At each study end point, six dogs were sacrificed, and euthanasia was performed with 2–3 cc Beuthanasia-D (Animal Health; TX, USA), followed by exsanguination or bilateral thoracotomy. Treatment sites and their surrounding bone and soft tissue were resected en bloc, fixed in 10% neutral buffered formalin for 7 days at room temperature and then stored in 70% ethanol.

### Micro-CT evaluation

Samples were imaged using a SkyScan 1176 (v1.1 Build 11; Bruker; MA, USA) at voltage and current set at 90 kV and 278 µA respectively, with 0.11 mm copper filter and 8.89 μm voxel image resolution. The region of interest (ROI) for analysis was defined as a hollow ring, 400 slices in height (3.55 mm from the implant apex) with a diameter of 0.63 mm and offset 0.2 mm from the surface of the dental implant to reduce metallic ring artifact (Supplementary Fig. 2). Images were reconstructed using NRecon software (with GPU acceleration v1.7.1.0; Bruker; MA, USA). The primary outcome measure for the µCT assessment of bone formation was percentage bone volume to tissue volume (BV/TV).

### Static and dynamic histomorphometry

Bone formation was further assessed by histomorphometric analyses of randomly selected Goldner’s trichrome-stained (static histomorphometry) and fluorescently-labelled (dynamic histormorphometry) tissue sections. Formalin-fixed tissue samples were dehydrated in ethanol baths of increasing concentrations, followed by defatting in xylene and infiltration and embedding in methyl methacrylate (MMA). Initial sectioning was performed in the bucco-lingual orientation using a low-speed diamond saw (Buehler; Switzerland). The sections were then ground to a thickness of approximately 50 μm using an EcoMet30 Auto-Polisher Grinder (ThermoFisher; MA, USA).

For static histomorphometry, tissue sections were stained with Goldner’s Trichrome, then mounted on glass slides and images were digitized using an Aperio ScanScope XT scanner and Aperio ImageScope software (Leica; Germany). Histomorphometry measurements were performed in an ROI defined as a 2D region with the same external dimensions as the micro-CT assessment (Supplementary Fig. 3). BioQuant Osteo Histomorphometric software (BioQuant; TN, USA) with customized human trabecular bone analysis protocol was used to quantify bone formation parameters. Percentage BV/TV was calculated as the sum of ROI measurements on both sides of the implant (buccal and lingual). Other parameters assessed include bone surface normalized to bone volume (BS/BV, mm^− 1^), trabecular number (Tb.N, mm^− 1^), trabecular separation (Tb.Sp, mm), osteoid volume to bone volume (OV/BV, %), osteoid surface to bone surface (OS/BS, %), osteoid width (O.Wi, µm), and number of osteoids per bone surface (N.Ob/BS, mm^− 1^).

For dynamic histomorphometric analysis of bone growth, animals in the 12-week group were subjected to intraperitoneal labeling with Alizarin Complexone (20 mg/kg; to label existing bone fronts) and Calcein (10 mg/kg; to label new bone fronts) fluorochromes at 14 and 7 days prior to sacrifice respectively. Following sacrifice, tissues were fixed and sectioned as previously described. A Nikon A1Si confocal microscope (Nikon; Japan) was used to capture Alizarin and Calcein fluorescence at the bone mineralization front on unstained tissue sections. Primary measurements of single and double-labeled bone surface area and inter-label distance were performed using the BioQuant Osteo 2019 (v199.96) Histomorphometric software (BioQuant; TN, USA) with customized human trabecular bone fluorescence analysis protocol. Mineral apposition rate (MAR, µm/day), and bone formation rate normalized to bone surface (BFR/BS, µm/day) were calculated based on the primary measurements.

### Collagen membrane barrier function

Treatment sites were also evaluated for the degree of epithelial ingrowth into bone defect space and membrane degradation assessed by the degree of resorption. The evaluation was performed by a qualified pathologist, blinded to the treatment group, using a semi-quantitative rubric adapted and modified from De Jong et al. [31, 32]. Details of the scoring system and definitions are provided in Supplementary Table 1.

### Statistical analysis

All data were imported into SPSS (v26; IBM; NY, USA) for descriptive statistical analyses comparing treatment groups at each endpoint with *p* < 0.05 was defined as statistically significant. Analysis of bone formation was performed on data generated by micro-CT of entire treatment sites and histomorphometric analysis of stained sections taken at varying levels within the treatment site. The normality of data was confirmed using Shapiro-Wilk’s test. Treatment group means were compared using an independent samples one-way analysis of variance method where equality of variances was not assumed (Welch’s ANOVA). If the difference between the means of the treatment groups were significant, post-hoc analysis was performed using pairwise Games-Howell tests. Epithelial ingrowth and membrane resorption were assessed using a semi-quantitative 5-point ordinal scale and statistical analyses performed using non-parametric, independent samples one-way analysis of variance (Kruskal-Wallis test). If a statistically significant difference was detected between treatment groups, post-hoc analysis was performed using pairwise Dunn’s tests with Bonferroni correction. Membrane resorptions were compared using a two-tailed Mann-Whitney U test.

## Results

### Animals

All eighteen animals recovered from surgery with no post-operative complications and were in good health until the scheduled sacrifice. There were no early deaths or clinical signs or symptoms of ill health throughout the study period. Submandibular lymph nodes were normal in contour, size, and shape.

### Micro-CT

**Fig. 2:**
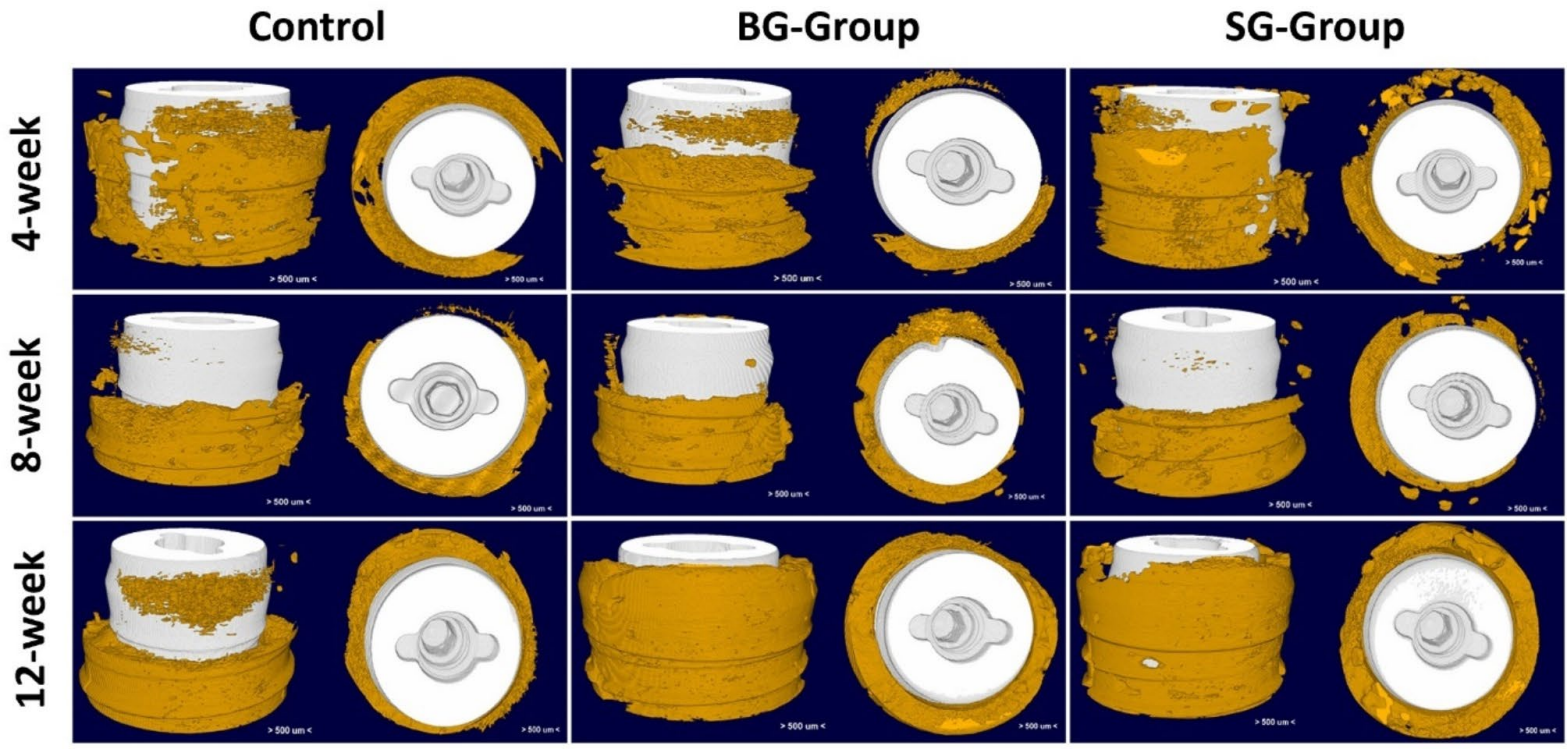
Representative 3D tomographic reconstruction of defect-implant site showing new bone formation (gold) around the implant at 4-, 8- and 12-weeks post-treatment

**Fig. 3:**
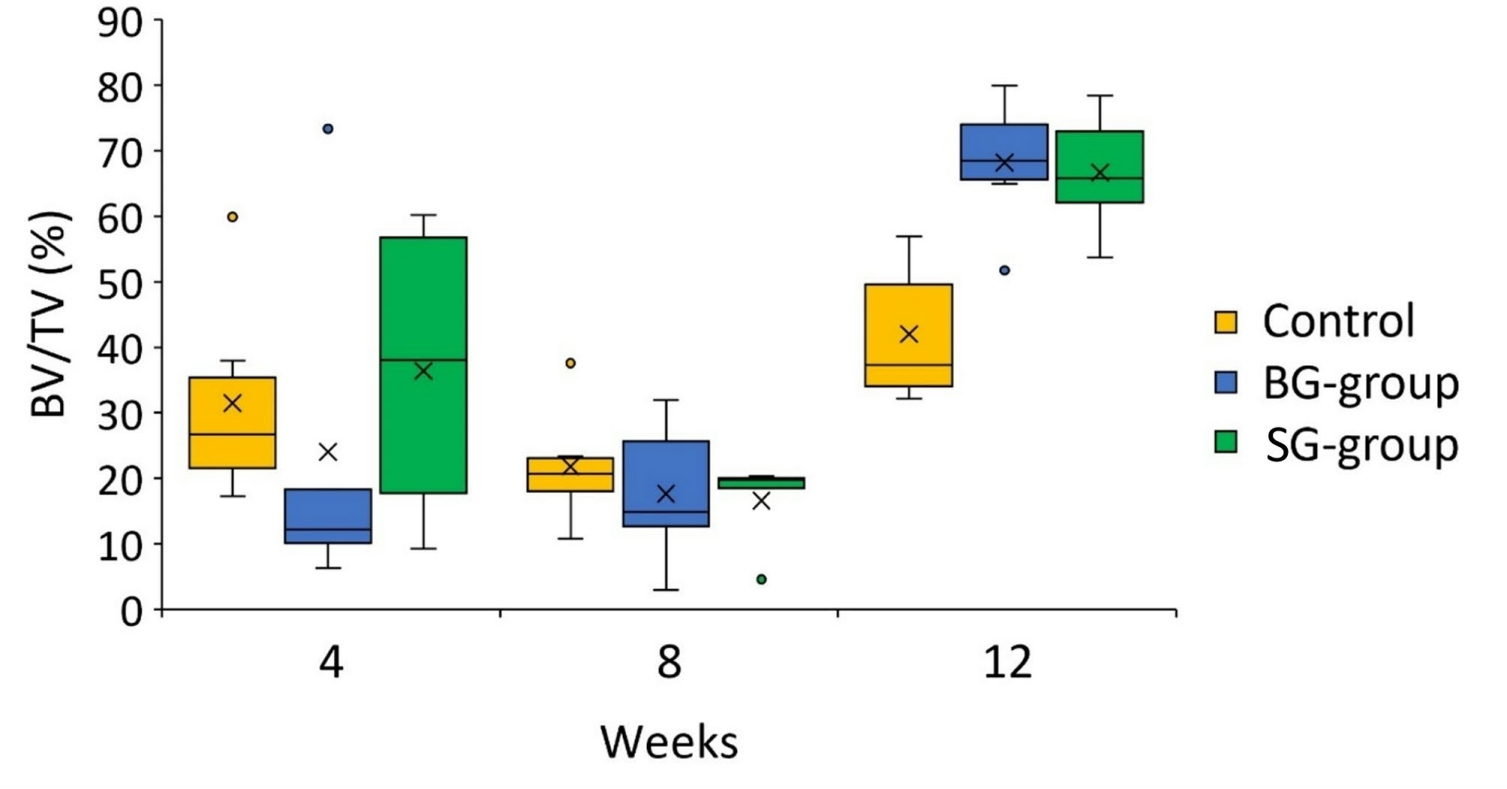
Assessment of bone formation using µCT. Percentage of bone volume normalized to tissue volume (BV/TV, %) comparison of study groups at 4-, 8- and 12-weeks post-treatment. Welch’s ANOVA with Games-Howell post hoc test; *p = 0.002, ** *p* = 0.003, and ns = no significant difference

All groups exhibited increased bone formation from 4-weeks to 12-weeks post-treatment (Figs. 2 and 3). At 4-weeks post-treatment, three-dimensional (3D) µCT reconstructed models showed similar levels of new bone formation between study groups. In all groups, bone fill surrounding the titanium dental implant was incomplete, with gaps clearly visible in the axial plane. Vertical regeneration was observed, with denser bone observed in the apical area of the ROI, but new bone formation did not extend coronally past the implant shoulder (Fig. 2). No significant difference in BV/TV was observed between groups with barrier membranes (BG and SG) to controls (Fig. 3).

At 8-weeks, more areas of consolidated, denser bone were observed in all group (Fig. 2). However, assessment of BV/TV revealed a slightly lower average amount of bone fill in each group compared to week 4, suggesting bone remodeling and turnover. Again, no statistically significant difference in mean BV/TV between groups was noted at 8-weeks post-treatment (Fig. 3).

Compared to 4- and 8-weeks post-treatment, significantly more and denser bone surrounding the implants that extended to the coronal implant surface was observed in both BG- and SG-groups at week 12 (Fig. 2). In comparison, less vertical bone fill in the control group was noted, but the new bone was dense and completely surrounded the implant in the axial plane (Fig. 2). There was a statistically significant difference between treatment group means at 12-weeks post-treatment (*p* = 0.006). Post-hoc tests further showed significantly higher BV/TV in BG-group (68.2 ± 9.76%, *p* = 0.002) and SG-group (66.7 ± 9.07%, *p* = 0.003) when compared to control animals (42.0 ± 10.78%) at 12 weeks. However, no statistically significant difference in bone formation was demonstrated between animals in the BG- and SG-groups (*p* = 0.96) (Fig. 3).

### Static histomorphometry

**Fig. 4:**
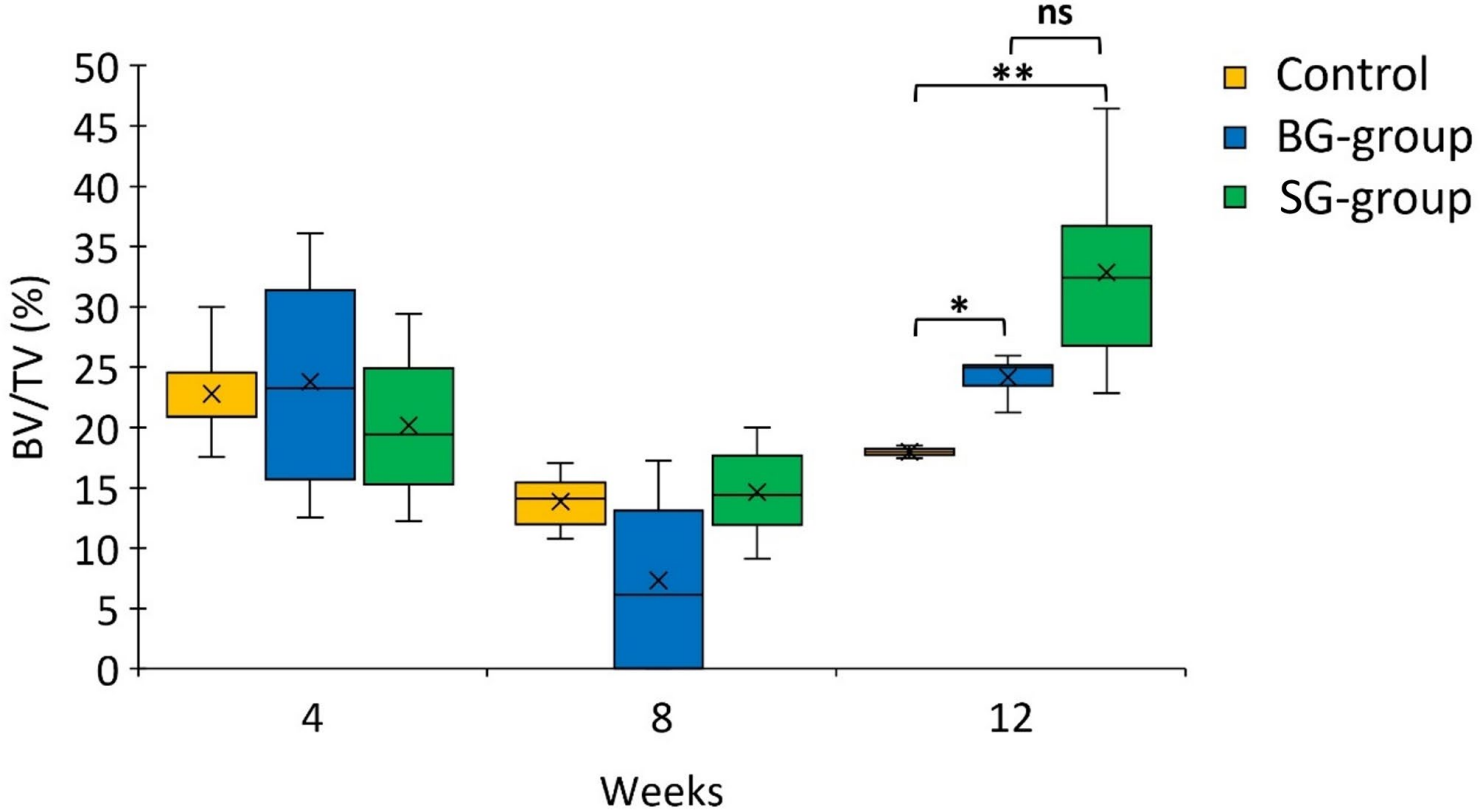
Histomorphometric assessment of bone formation in study groups at 4-, 8-, and 12-weeks post-treatment. Percentage BV/TV comparison between study groups with Welch’s ANOVA and Games-Howell post-hoc test; * *p* = 0.004, ** *p* = 0.005, and ns = no significant difference

At week 4, there was no significant difference in BV/TV or BS/TV between SG-, BG- and control groups (Fig. 4). All other bone formation parameters were also similar between groups with no statistically significant difference observed in any of the parameters (Table 1). Consistent with micro-CT assessment of bone formation, similar results were observed at 8-weeks post-treatment (Table 2), although lower and larger variation of BV/TV was observed in BG group (Fig. 4), and no significant difference was demonstrated between treatment groups. No significant difference were noted between treatment groups in other parameters including BS/TV, and bone architecture parameters Tb.N, Tb.Sp, and O.Wi. However, the number of osteoblastic cells per bone surface (N.Ob/BS) was significantly lower in the BG-group when compared to control groups (*p* = 0.05) (Table 2). Together these results suggested that interrupted or delayed bone formation was observed in the BG group, and bone formation may have occurred earlier in the SG group.

**Table 1:** Static histomorphometry assessment of bone formation parameters at 4-weeks post-treatment

Parameter	Control	BG	SG	ANOVA (*p*-value)
*N* (sections)	6	4	6	
BV/TV (%)	25.4 ± 7.6	23.8 ± 11.0	20.2 ± 6.8	0.51 (ns)
BS/BV (mm)	4.3 ± 1.5	4.2 ± 0.5	4.2 ± 1.6	0.98 (ns)
Tb.N (mm^− 1^)	0.6 ± 0.2	0.6 ± 0.1	0.5 ± 0.2	0.70 (ns)
Tb.Sp (mm)	0.8 ± 0.2	0.9 ± 0.5	1.1 ± 0.6	0.50 (ns)
OV/BV (%)	3.0 ± 0.8	2.6 ± 1.4	2.3 ± 1.9	0.74 (ns)
OS/BS (%)	60.1 ± 11.3	54 ± 21.1	46.3 ± 26.9	0.54 (ns)
O.Wi (µm)	11.2 ± 1.37	10.2 ± 2.3	9.9 ± 2.1	0.47 (ns)
N.Ob/BS (mm^− 1^)	18.8 ± 11.0	21.5 ± 2.7	14.2 ± 10.5	0.32 (ns)

Values are mean ± SD; N = number of sections; BV/TV = bone volume/tissue volume; BS/BV = bone surface/bone volume; Tb.N = trabecular number; Tb.Sp = trabecular seperation; OV/BV = osteoid volume/bone volume; OS/BS = osteiod surface/bone surface; O.Wi = osteiod width; N.Ob/BS = number of osteoid/bone surface; ns = no significant difference

**Table 2:** Static histomorphometry assessment of bone formation parameters at 8-weeks post-treatment

Parameter	Mean ± SD			*P*-value			
	Control	BG	SG	ANOVA	Control vs. SG	Control vs. BG	BG vs. SG
*N* (sections)	6	5	7				
BV/TV (%)	13.9 ± 2.4	7.3 ± 7.8	15.0 ± 3.9	0.21 (ns)			
BS/BV (mm)	3.5 ± 1.6	1.8 ± 1.8	3.5 ± 1.5	0.26 (ns)			
Tb.N (mm^− 1^)	0.4 ± 0.2	0.2 ± 0.2	0.4 ± 0.2	0.23 (ns)			
Tb.Sp (mm)	1.9 ± 0.8	1.6 ± 2.0	2.1 ± 2.0	0.90 (ns)			
OV/BV (%)	1.2 ± 0.7	0.5 ± 0.6	1.8 ± 1.2	0.08 (ns)			
OS/BS (%)	35.8 ± 11.6	13.4 ± 18.8	42.9 ± 16.5	0.06 (ns)			
O.Wi (µm)	9.9 ± 2.3	3.9 ± 5.3	11.0 ± 2.2	0.07 (ns)			
N.Ob/BS (mm^− 1^)	17.7 ± 11.1	2.6 ± 4.8	11.5 ± 7.8	0.024	0.50 (ns)	0.05	0.08 (ns)

Values are mean ± SD; N = number of sections; BV/TV = bone volume/tissue volume; BS/BV = bone surface/bone volume; Tb.N = trabecular number; Tb.Sp = trabecular seperation; OV/BV = osteoid volume/bone volume; OS/BS = osteiod surface/bone surface; O.Wi = osteiod width; N.Ob/BS = number of osteoid/bone surface; ns = no significant difference

In contrast to the 4- and 8-weeks post-treatment assessments, significantly higher BV/TV was demonstrated in BG- and SG-groups at 12 weeks post-treatment compared with controls, but no difference between the two membrane groups. This result was consistent with the observations shown in µCT assessment. On the other hand, BV/TV in control groups was marginally increased from week 8 but overall bone formation was less than initially observed at 4 weeks (Fig. 4; Table 3). Assessment of bone microarchitectural parameters showed that animals in SG-group exhibited significantly higher Tb.N, O.Wi and lower Tb.Sp, suggesting more favorable mature bone structure. A significant increase in the number of osteoblasts on bone surface was seen in SG-group in comparison to the control group consistent with higher bone formation in SG-group (Table 3). No significant difference in any bone microarchitectural parameters were noted between BG- and SG-groups. These data suggest that the use of collagen membranes facilitates bone formation in GBR, with Striate + providing significantly better bone formation than controls and trend towards superior outcomes than animals treated with Bio-Gide at 12 weeks (Table 3).

**Table 3:** Static histomorphometry assessment of bone formation parameters at 12 weeks post-treatment

Parameter	Mean ± SD			*P*-value			
	Control	BG	SG	ANOVA	Control vs. SG	Control vs. BG	BG vs. SG
*N* (sections)	3	7	8				
BV/TV (%)	18.2 ± 0.6	26.3 ± 4.0	31.5 ± 8.1	< 0.001	0.005	0.004	0.28 (ns)
BS/BV (mm)	2.6 ± 0.5	3.0 ± 0.4	3.3 ± 0.8	0.26 (ns)			
Tb.N (mm^− 1^)	0.3 ± 0.1	0.4 ± 0.1	0.5 ± 0.1	0.017	0.012	0.06 (ns)	0.22 (ns)
Tb.Sp (mm)	1.7 ± 0.3	1.0 ± 0.2	0.8 ± 0.3	0.013	0.027	0.07 (ns)	0.11 (ns)
OV/BV (%)	0.7 ± 0.2	0.7 ± 0.6	1.1 ± 0.3	0.08 (ns)			
OS/BS (%)	31.2 ± 2.2	24.8 ± 14.7	29.6 ± 4.6	0.48 (ns)			
O.Wi (µm)	9.1 ± 0.7	9.1 ± 2.6	11.0 ± 1.3	0.04	0.038	0.99 (ns)	0.25 (ns)
N.Ob/BS (mm^− 1^)	1.5 ± 1	7.0 ± 5.6	7.8 ± 4.6	0.007	0.014	0.09 (ns)	0.94 (ns)

Values are mean ± SD; N = number of sections; BV/TV = bone volume/tissue volume; BS/BV = bone surface/bone volume; Tb.N = trabecular number; Tb.Sp = trabecular seperation; OV/BV = osteoid volume/bone volume; OS/BS = osteiod surface/bone surface; O.Wi = osteiod width; N.Ob/BS = number of osteoid/bone surface; ns = no significant difference

### Dynamic histomorphometry

Alizarin/calcein double-labelling was carried out to assess in vivo bone formation rate 12 weeks post-treatment (Supplementary Fig. 4). There were no bone abnormalities observed in all samples. Dynamic histomorphometry showed similar active bone formation and mineralization denoted by bone formation rate (BFR/BS) and mineral apposition rate (MAR) respectively, in all treatment groups (Table 4).

**Table 4:** Dynamic histomorphometric assessment of bone formation at 12 weeks post-treatment

Parameter	Control	BG	SG	ANOVA (*p*-value)
MAR (7d interval)	0.38 ± 0.22	0.39 ± 0.20	0.39 ± 0.44	ns
BFR/BS	2.56 ± 1.46	1.85 ± 1.18	1.91 ± 2.16	ns

Values are mean ± SD; ns = no significant difference; MAR = mineral apposition rate; BFR/BS - bone formation rate to bone surface

### Bone formation in descriptive histology

**Fig. 5:**
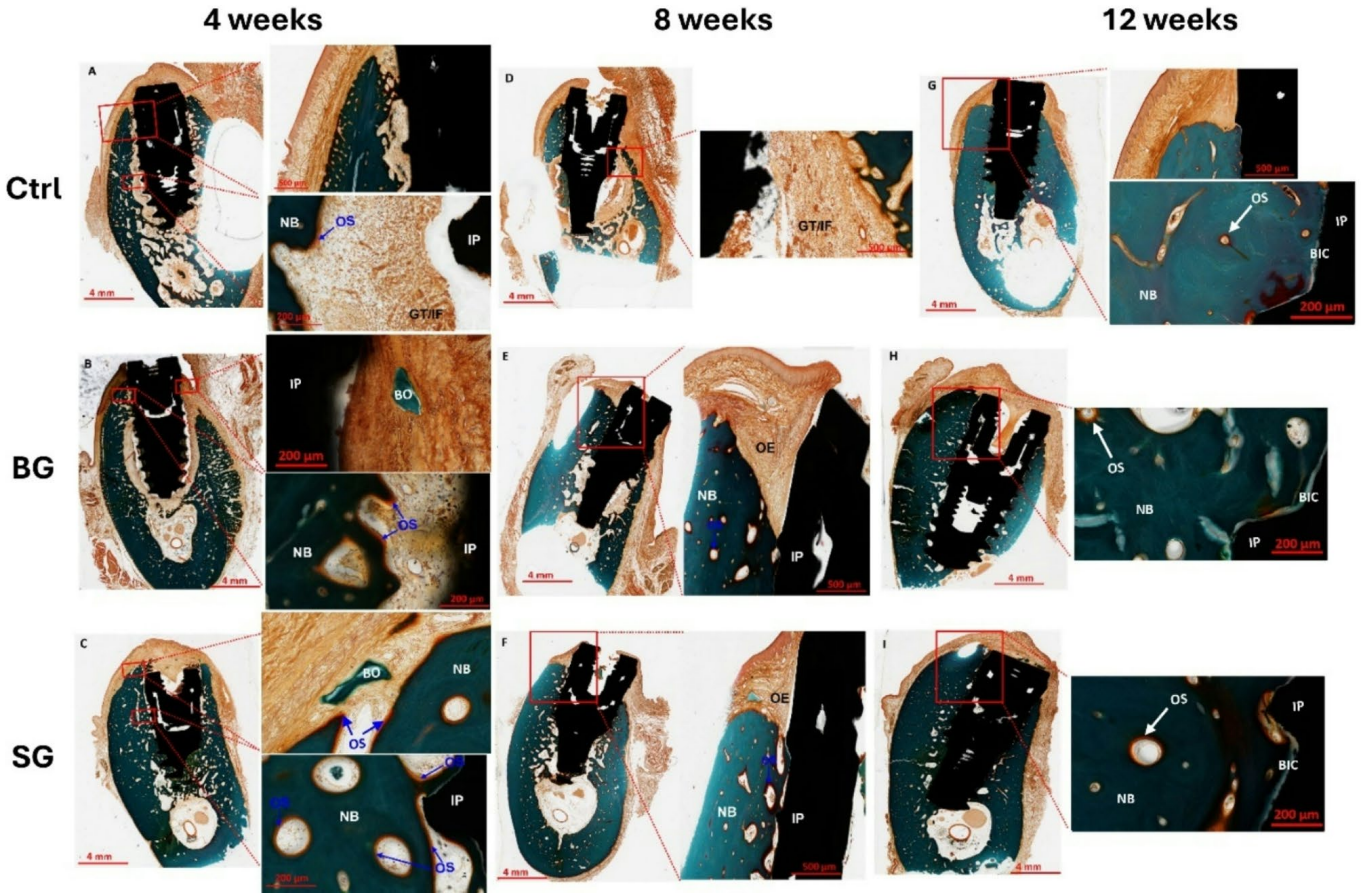
Histological assessment of bone regeneration using Goldner Trichrome stained sections at 4-, 8- and 12-weeks post-treatment. (**A**, **B** and **C**) At week 4: **(A)** In the control group, little bone formation with granulation tissue and heavy inflammatory infiltrate was observed. **(B)** In the BG-group, significant bone regeneration was observed, with some epithelial invasion into the defect and the presence of granulation tissue. Less inflammation compared to control group was noted. **(C)** In SG-group, abundant bone regeneration was observed throughout the bone socket, with some granulation tissue. Inflammatory reaction was less than control group. (**D**, **E** and **F**) At week 8: **(D)** In the control group, poor bone formation with granulation tissue and heavy inflammatory infiltrate present in defect site. **(E)** Significant bone regeneration was demonstrated in BG-group with good bone-implant contact observed in some samples. **(F)** Abundant bone regeneration was observed around the implant in the majority of samples from SG-group. (**G**, **H** and **I**) At week 12: **(G)** In the control group, significant bone regeneration with good bone-implant contact was observed. **(H)** Thick and dense mature bone surrounds the implant in BG-group with good bone-implant contact observed. **(I)** Abundant mature bone was observed around the implant in all samples from SG-group with excellent bone-implant contact. NB– new bone; IP– implant; OS– osteoid; GT– granulation tissue; IF– inflammatory cells; BO– Bio-Oss material; OE– oral epithelium; BIC– bone-to-implant contact

In Goldner Trichrome stained sections, treatment sites from control group at week 4 showed less mature bone formation as compared to BG- and SG-groups and the newly formed bone matrix did not reach the level of the implant shoulder (Fig. 5A, NB). Most newly formed bone was thin trabecular bone matrix around the base of the implants. While oral epithelium and underlying connective tissue covered the socket and the implant, infiltration of the underlying connective tissue with inflammatory cells and epithelium was evident in some areas between bone and titanium implant (Fig. 5A). Bone-to-implant contact was not ideal as bone formation occurred distally to the implant surface in most samples, with gaps between bone and implant interface frequently observed. In the mid portion of implant, unresorbed Bio-Oss material with granulation tissue, giant cells and inflammatory cells were abundant between the implant and newly formed bone (Fig. 5A, GT/IF).

No significant differences in morphological features of bone formation and tissue structure between BG- and SG-group was noted at 4 weeks post-treatment (Fig. 5B and C). In both barrier membrane groups, bi-directional new bone formation was observed, and all sockets were filled with newly formed bone. Oral epithelium and underlying connective tissue covered the bone socket and the implants. The newly formed bone was well integrated with the existing mandibular bone matrix and indistinguishable from existing socket bone wall (Fig. 5B and C, NB). The majority of the newly formed bone matrices were well-connected thick trabecular bones. Vertical bone regeneration was observed up to the level of the implant shoulder. In the defect between the socket wall and titanium implant, active bone formation was evident due to the presence of abundant osteoid matrix with granulation tissue (Fig. 5B and C, NB). In some sections, Bio-Oss material were observed in the bone defect (Fig. 5B and C, BO).

At 8 weeks, treatment sites from the control group showed incomplete bone formation surrounding the implant. In some areas, there was insufficient coverage of bone matrix between the implant and soft tissue (Fig. 5D). Although oral epithelium and underlying connective tissue covered the socket and the implant, overgrowth of gingival tissue onto the implant surface was evident. At the base of implant, there was interspersed fibrotic granulation tissue and inflammatory cells in the socket between the implant and bone (Fig. 5D, GT/IF). Bone to implant contact was limited and did not extend past the level of the implant shoulder.

In the BG- and SG-groups, histological features at 8 weeks were very similar to 4 weeks but more bone remodeling was observed (Fig. 5E and F). No significant differences in morphological features of bone formation and tissue structure were observed between the BG- and SG-groups. Active bone formation was predominantly seen at the base of the implant and extended to the level of the shoulder of the implants (Fig. 5E and F, NB). Oral epithelium and underlying connective tissue completely covered the socket and the implant (Fig. 5E and F, OE). Newly formed bone surrounding the implant was trabecular in appearance with active osteoid surface (Fig. 5E and F, OS).

At 12 weeks post-treatment, consolidation of woven bone into mature bone was observed in control groups, with good bone-to-implant contact (Fig. 5G, BIC and NB). However, less bone was observed compared to barrier membrane groups, with crest height in control samples remaining below the level of the implant shoulder. Some Bio-Oss materials were visible in the vicinity of the implant in control groups. In contrast, there were thick and dense mature bone surrounding the implant in both the BG- and SG-groups (Fig. 5H and I). Trabecular structure of bone at the surface of implants were replaced by dense trabecular plate, increasing surface contact between implants and new bone (Fig. 5H and I; BIC). In the BG-group, 16% of samples examined exhibited mature bone above the level of the implant shoulder, extending to the coronal surface of the implant. In comparison, 50% of samples from SG-group showed mature bone overgrowth above the implant shoulder and around the coronal surface of the implant. Oral epithelium and underlying connective tissue completely covered the socket and implant. The entire socket area was occupied by newly formed bone. Bone marrow cavities were established and were filled with abundant normal marrow cells and microcapillaries. No residual Bio-Oss materials were observed in the vicinity of the implant. No significant differences in histological features of bone formation and tissue structure between BG- and SG-group were noted.

### Osteoblast and osteoclast activities

**Fig. 6:**
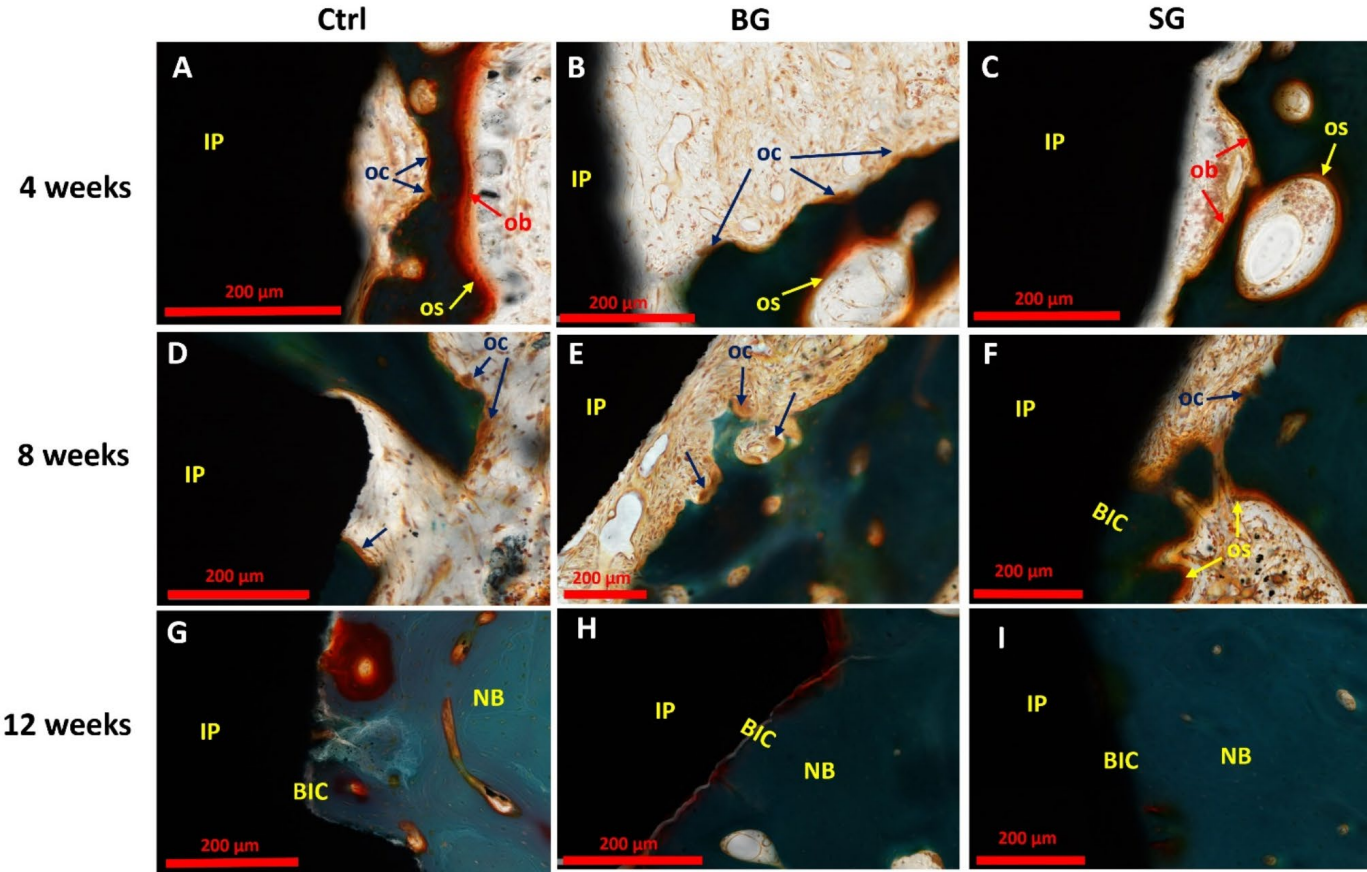
Histological assessment of osteoblast and osteoclast activities using Goldner Trichrome stained sections at 4-, 8- and 12-weeks post-treatment. (**A**, **B** and **C**) At week 4: **(A)**. Osteoblasts with osteoids can be observed on one side of the bony islands and osteoclasts are present on the other side. **(B)** Osteoclast activity and osteoid deposition are prevalent in BG-group at week 4. **(C)**. Osteoblasts and osteoid deposition are prevalently observed in SG-group at week 4. (**D**, **E** and **F**) At week 8: Active osteoclasts are prevalent in all three groups, while SG-group **(F)** displayed early signs of bone-to-implant contact. (**G**, **H** and **I**) At week 12: Good bone-to-implant contact in all groups at week 12. NB– new bone; IP– implant; OS– osteoid; OB– osteoblasts; OC– osteoclasts; BIC– bone-to-implant contact

At week 4, all three study groups exhibited insufficient bone-to-implant contact, and osteoids can be observed showing active bone formation (Fig. 6, OS/yellow arrows). In the control group, osteoblasts with osteoids can be observed on one side of the bony islands (Fig. 6A, OS/OB) and osteoclasts were present on the other side (Fig. 6A, OC). Histological assessments revealed a prevalence of osteoclast activity and osteoid deposition in the BG-group (Fig. 6B, OC), contrasting with a notable increase in osteoblasts and osteoid deposition in the SG-group (Fig. 6C, OS/OB).

By week 8, an increased level of resorption persisted across all groups with the presence of active osteoclasts (Fig. 6D-F, OC), consistent with the reduced BV/TV ratios in micro-CT analysis and static histomorphometry. This indicated the commencement of bone remodeling process. Interestingly, the SG-group displayed early signs of bone-to-implant contact, reflecting an accelerated osteogenic response (Fig. 6F, BIC).

At week 12, a restorative trend was observed across all experimental groups, culminating in the establishment of robust bone-to-implant interfaces, signifying a return to physiological homeostasis in the context of implant osseointegration (Fig. 6G-I). These nuanced temporal dynamics underscore the intricate interplay between osteoclastic and osteoblastic activities, ultimately influencing the spatiotemporal patterns of bone regeneration and implant integration.

### Barrier membrane function – epithelial invasion and membrane resorption/remodeling

**Fig. 7:**
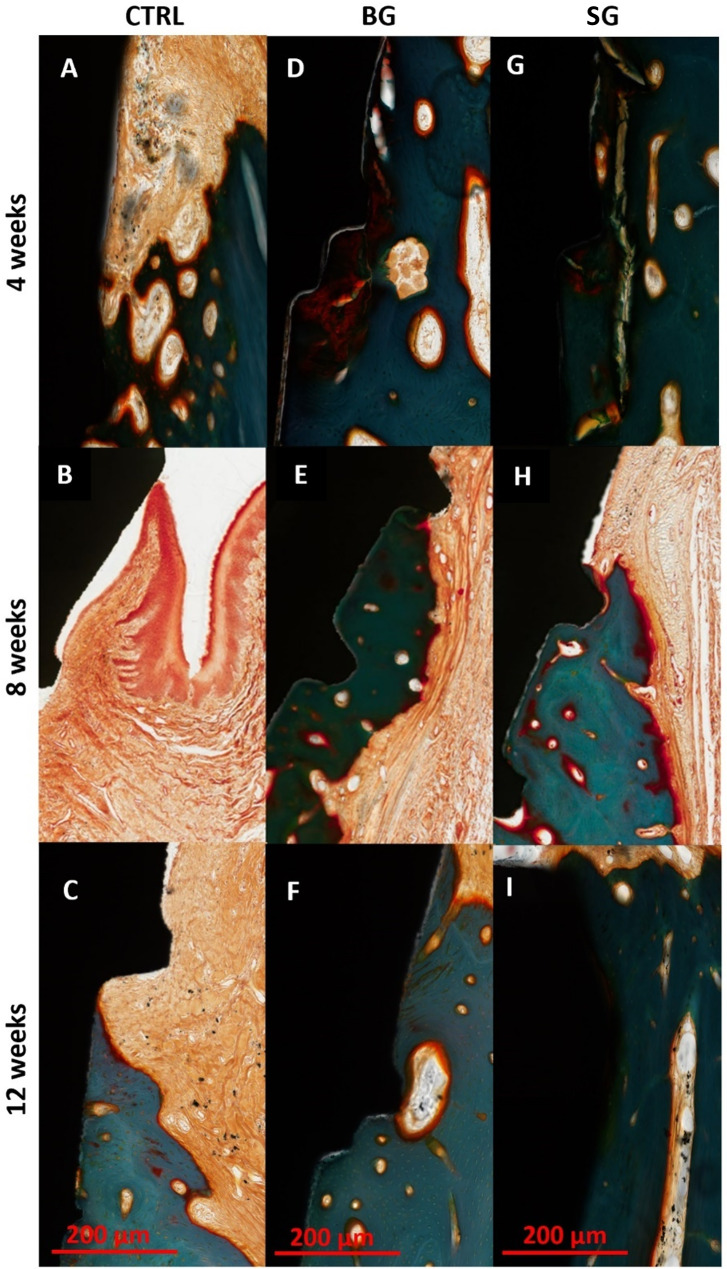
Representative images of epithelial ingrowth at week 4, 8 and 12 (Goldner’s trichrome, 200x magnification). **A**, **B** and **C**– Control group without collagen membrane. Ingrowth epithelium layer forms a sulcus adjunct to the shoulder of implant and continues ingrowth down below the shoulder of implant **(B)**. Connective tissue was infilled in the region between epithelium and bone matrix. **D**, **E** and **F**– BG-group. Dense connective tissue on the surface bone matrix. Some minor degree of epithelial ingrowth at week 4 **(D)**. **G**, **H** and **I**– SG-group. Dense connective tissue on the surface bone matrix. No epithelial ingrowth into the vicinity of implant surface

In the control group without the use of collagen barrier membranes, moderate epithelial layer with reticular connective tissue ingrowth to the vicinity of implant was observed 4 weeks post-treatment, and becoming more prominent at 8-weeks (Fig. 7A and B). By week 8, ingrowth epithelial layers formed a sulcus adjacent to the shoulder of the implants and continued to invade down below the shoulder of the implants. In regions where epithelial ingrowth into the socket was observed, connective tissue infill was seen in the region between the epithelium and bone matrix (Fig. 7B). Continued epithelial ingrowth was observed in the control samples at week 12 but was not as obvious as at week 8 (Fig. 7C). Samples from BG-group showed some degree of epithelial ingrowth at 4- and 8-weeks post-treatment (Fig. 7D and E) but was not evident by 12-weeks (Fig. 7F). In contrast, little epithelial invasion was noted at week 4 and none was observed in any of the samples from the SG-group at week 8 and 12 (Fig. 7G-I).

**Fig. 8:**
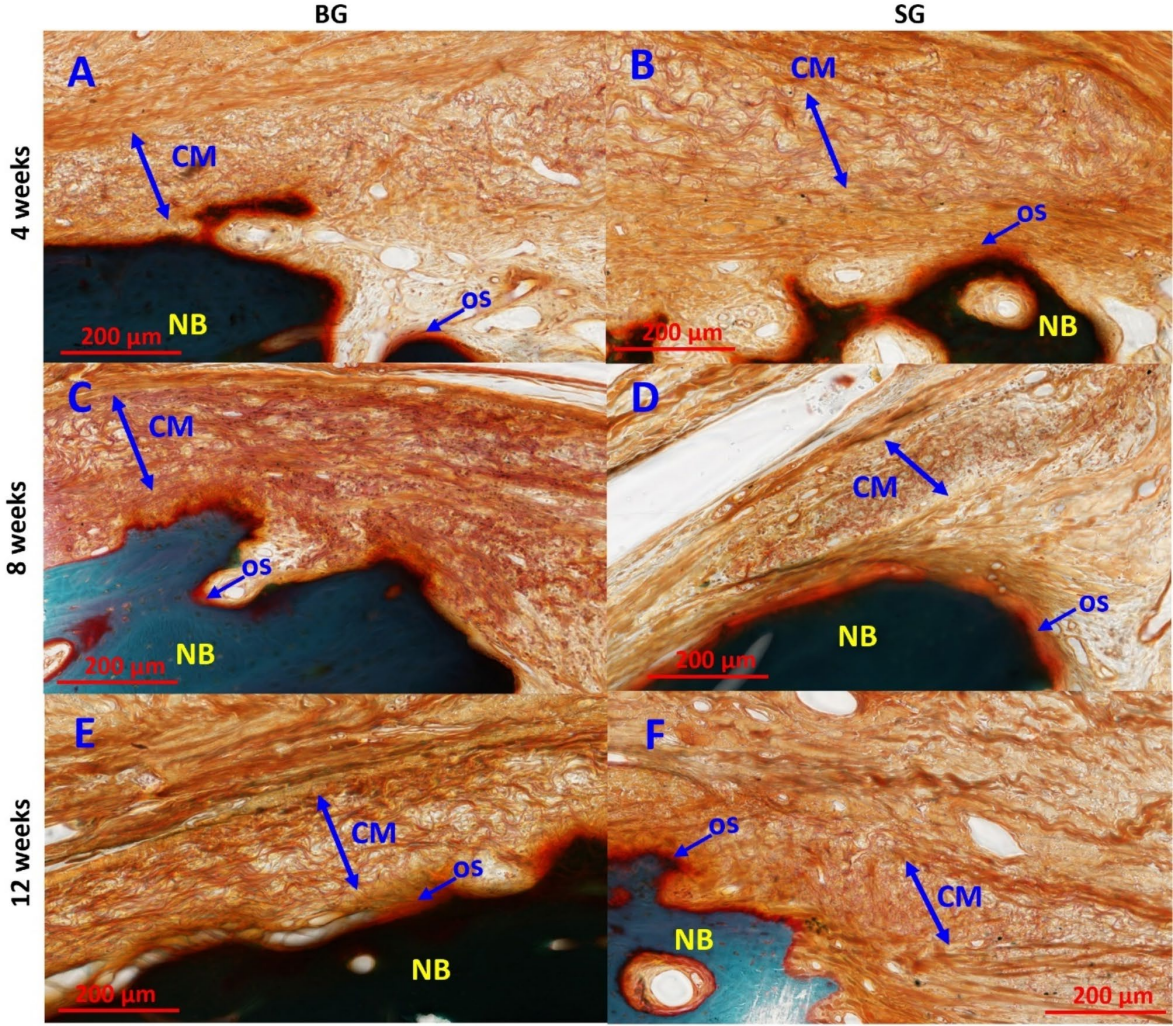
Representative histological assessment of collagen membrane resorption. (**A**, **C**, and **E**) BG-group and (**B**, **D**, and **F**) SG-group at 4-, 8-, 12-week post-treatment respectively. Double arrows demarcate collagen membrane from surrounding tissue and implant, showing reduced thickness due to resorption and remodeling of membranes overtime. Goldner’s trichrome stained sections imaged at 200× magnification; CM– collagen membrane; OS– osteoid; and NB– natural bone mineral

**Fig. 9:**
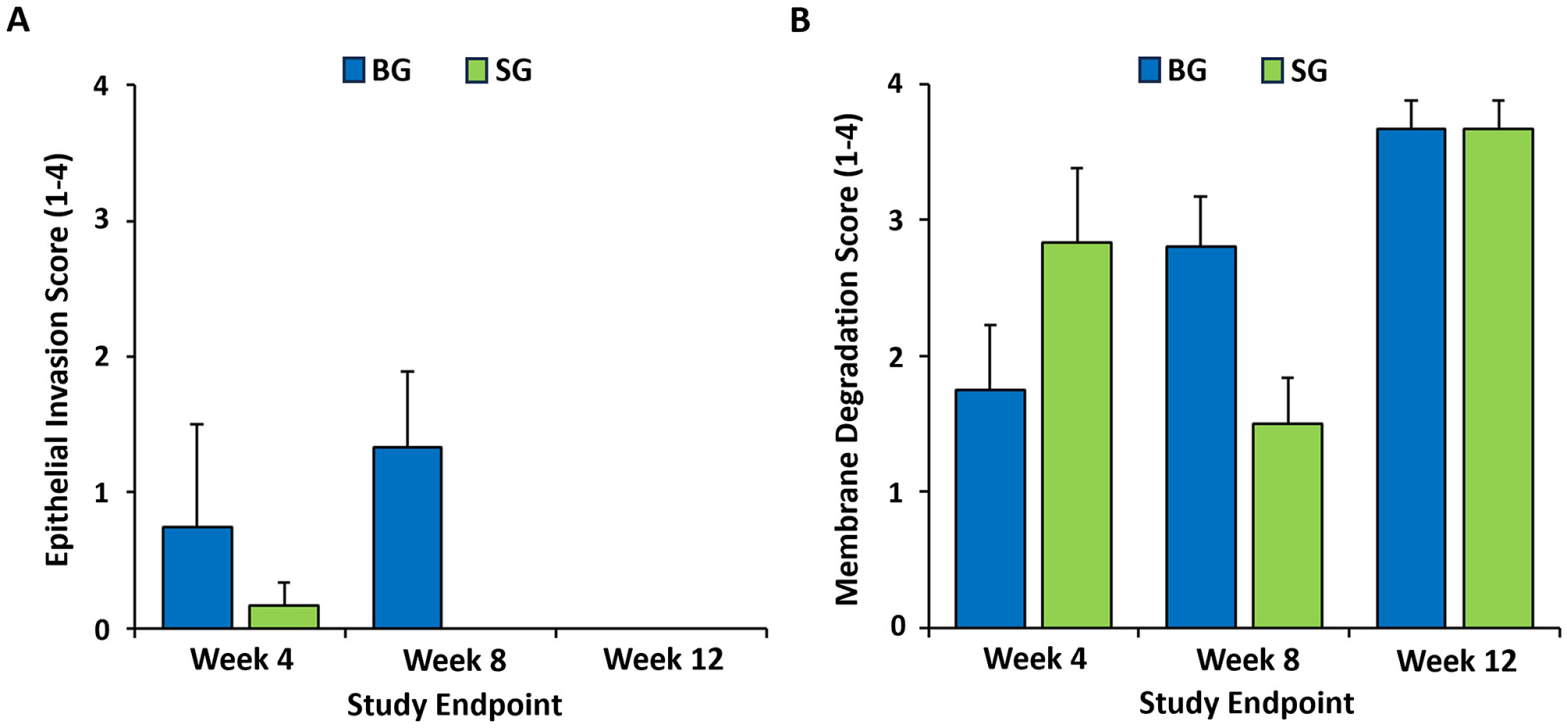
Assessment of barrier membrane function. Semi-quantitative scoring of **(A)** epithelial invasion and **(B)** membrane degradation of Bio-Gide and Striate + in accordance with ISO 10993:6 at 4-, 8-, and 12-weeks post-treatment. Data presented as mean ± SD

In terms of membrane degradation, at 4-weeks post-treatment, both Bio-Gide and Striate + were evident in the region containing bone matrix and reticular tissue underneath of epithelial membrane layer (Figs. 8A and B; CM). Membrane discontinuity in some of the samples of both collagen membranes could have contributed to the minor epithelial invasion seen in both groups at this timepoint (Fig. 7D and G). No foreign body giant cells were noted near the vicinity of the membranes. By 8-weeks, Bio-Gide was more markedly resorbed with small discontinuous remnants, and almost completely resorbed by 12-weeks with speckled distribution of small fragments throughout the connective tissue layers (Fig. 8C and E). Moderate levels of resorption and remodeling was noted for Striate + in the SG-group samples at 8-weeks and was well-integrated and remodeled into surrounding connective tissue by 12-weeks (Fig. 8D and F). In both membrane groups, bone defect space was almost completely filled with new bone by 12-weeks. Semi-quantitative assessment showed no significant difference in epithelial ingrowth score (Fig. 9A) and membrane resorption score (Fig. 9B) between BG- and SG-groups.

## Discussion

The objective of this study was to evaluate the effectiveness of Striate+, a novel collagen membrane, for use in guided bone regeneration including the capability to enhance bone formation and barrier characteristics. Our study showed that use of either Striate+ (SG) or Bio-Gide (BG) barrier membranes for GBR can achieve significantly higher bone volume when compared to the controls without barrier membrane. Interestingly, assessment of bone microarchitectural parameters showed that animals in SG-group exhibited significantly higher Tb.N, O.Wi and lower Tb.Sp, suggesting more favorable mature bone structure. Histological assessment showed that SG-group displays early signs of bone-to-implant contact at 8 weeks. Our study indicated the effectiveness of Striate + in GBR.

This comprehensive assessment, which included micro-CT, static and dynamic histomorphometry and histological examination, investigated GBR around the implant and surrounding soft tissue. Micro-CT measurement of bone volume (BV/TV) in a defined region of interest around the upper third of the dental implants showed substantial bone formation at 4 weeks with no significant difference between groups. Bone volumes were reduced at week 8 as remodelling occurred but by week 12, bone volume had significantly increased in all groups [33, 34]. At week 12, significantly higher bone volume was observed in the Striate + or Bio-Gide treated sites than control sites. This trend is consistent with previous studies using non-crosslinked collagen membranes [35–38]. Another study on alveolar contour after guided bone regeneration in beagle dogs also reported that after 16 weeks, significant gains in bone contour was observed in test groups using collagen membranes, in comparison with control group [39]. Although there was no difference in bone formation between Bio-Gide and Striate + in GBR, it appears that use of Striate + can achieve earlier bone regeneration as compared to Bio-Gide in a canine GBR model.

Multiple studies have shown that microCT along with histomorphometric analysis has been a reliable method to evaluate bone formation and remodeling in GBR [8, 11, 40]. In this study, static histomorphometry analysis further confirmed micro-CT results, demonstrating that use of a collagen membranes resulted in significantly higher BV/TV, compared to control at week 12. This is consistent with previous studies indicating that 12 weeks (or 3 months) post-operation is a sufficient time period to observe a difference in bone volume [41–43]. Interestingly it is noted that the number of osteoblastic cells per bone surface in the BG-group but not SG group was significantly lower when compared to control groups at 8 weeks. However, there was no differences in the number of osteoblasts per bone surface at 12 weeks between the groups. Together these results may suggest differences in initiating bone formation between the collagen membranes used.

Dynamic histomorphometry studies further showed that the bone formation rate is comparable between groups. The results showed that there was no alteration of bone mineralization or crystal deposition process between groups. As the split-mouth model was used in this study, these results also suggest that the bone formation rate is comparable between treatment sites [44].

Histological evaluation of bone tissue in GBR-treated sockets at 4 weeks revealed the formation of new bone that was well integrated with the existing mandibular bone matrix. Greater vertical bone regeneration was observed with Striate+, up to the level of the implant shoulder at 4 weeks, compared to control. Coverage of the sockets and implants with oral epithelium and underlying connective tissue increased over time with better coverage in Striate + and Bio-Gide sites than in control group. At 12 weeks, formation of thick and dense mature bones surrounding the titanium implant with good bone-to-implant contact was seen in both Striate + and Bio-Gide groups; bone marrow cavities had been established and were filled with abundant normal marrow cells and microcapillaries. This is similar to the results of previous studies stating that after 8 weeks, collagen membranes had integrated uneventfully with surrounding tissues and obtained satisfactory osseointegration [20, 21, 45]. Jin et al. also described in his report that the surrounding tissues were fully integrated and matured, forming dense connective tissue that resembled periosteum as the membranes were eventually replaced by connective tissue, which is similar to what we observed in our study [21].

Several studies have indicated that use of collagen membranes as a barrier structure is capable of preventing soft tissue invasion and thereby increase the height of bone formation to the shoulder of implant [45–47]. A recent study also showed that both Striate + and Bio-Gide membranes can block 0.2–16.4 μm beads from passing through them [12]. In our study, we showed that epithelial ingrowth into the bone defect occurred without use of barrier membranes. Bornstein et al. suggested that as the collagen barrier membrane preserved the space made during surgery and clearly distinguished the bone/marrow cavity from the outer gingival tissues, it certainly had a significant impact on bone regeneration, demonstrated by more complete osseous healing [46]. Another study on guided regeneration in bone defects in dogs demonstrated that the barrier features of collagen membranes even allowed a thin regenerated cementum layer to develop on the dentine surface without the interference of unwanted gingival tissues [47].

Previous studies have shown that the resorption kinetics of collagen membranes vary depending on the experimental model. Complete degradation of collagen membrane has been reported as early as 4 weeks in studies where no bone grafting material or implant was used [48]. In published GBR studies of Bio-Gide with an implant and Bio-Oss, complete degradation occurred between 8 and 16 weeks [49–50]. The time frame of collagen membrane resorption in this study is consistent with published studies, resorption of the collagen barrier membranes had commenced at week 4 and was almost complete by week 12 with small membrane fragments visible in some sections. Friedmann et al. demonstrated in their study that collagen membranes with a prolonged resorption/barrier profile are more efficient in supporting the bone regeneration process [51]. Thus, it is suggested that both membranes exhibit adequate space maintenance ability and favorable barrier characteristics to prevent unwanted epithelial and inflammatory infiltration.

## Conclusion

The findings of this study demonstrated that Striate + collagen membrane can significantly enhance bone regeneration and prevent unwanted epithelial infiltration. These results provided a foundation for further clinical investigations to assess its long-term efficacy in various clinical settings.

## Electronic supplementary material

Below is the link to the electronic supplementary material.

Supplementary Material 1

Supplementary Material 2

Supplementary Material 3

Supplementary Material 4

Supplementary Material 5

## Data Availability

No datasets were generated or analysed during the current study.
