# Supplementary material for: The efficacy of a novel porcine-derived collagen membrane on guided bone regeneration: a comparative study in canine model
Source: BMC Oral Health. 2025 May 29;25:850. doi: 10.1186/s12903-025-05930-6 (PMC12123806; doi:10.1186/s12903-025-05930-6)
Supplement: Supplementary file 1 — Supplementary Material 1 [file 12903_2025_5930_MOESM1_ESM.docx]

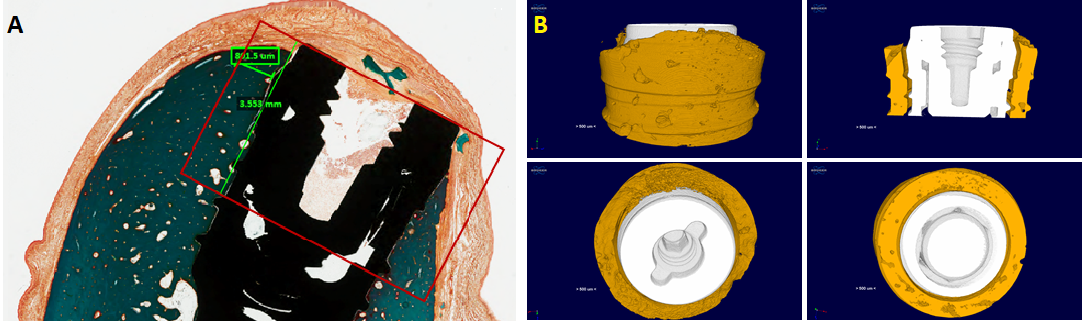


**Supplementary Figure 3.** The region of interest (ROI) for static histomorphometry was defined by selecting a regular box of 3.55mm height from the implant apex with a diameter of 0.8mm, no offset from the bone surface to the dental implant was selected.
