# Supplementary material for: The efficacy of a novel porcine-derived collagen membrane on guided bone regeneration: a comparative study in canine model
Source: BMC Oral Health. 2025 May 29;25:850. doi: 10.1186/s12903-025-05930-6 (PMC12123806; doi:10.1186/s12903-025-05930-6)
Supplement: Supplementary file 2 — Supplementary Material 2 [file 12903_2025_5930_MOESM2_ESM.docx]

*
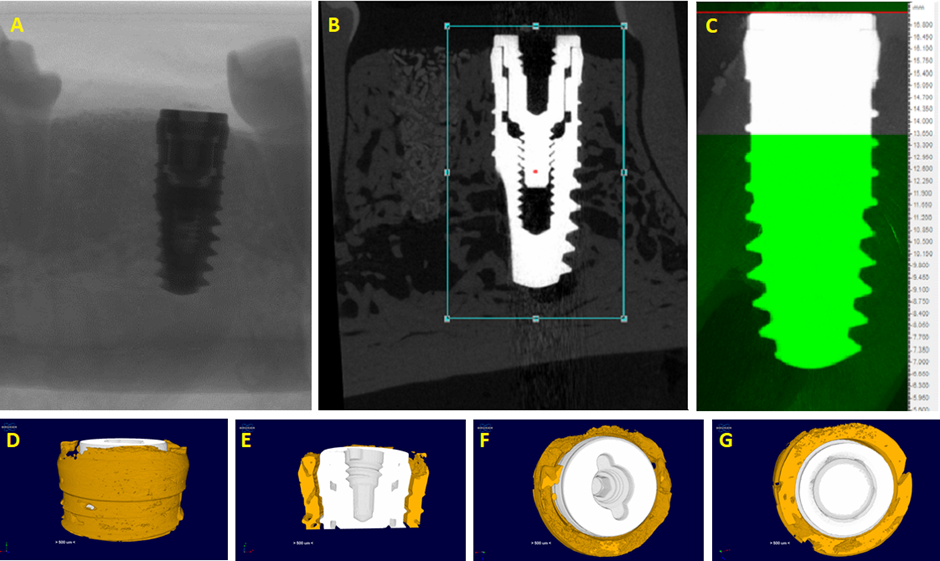
*

Supplementary Figure 2. MicroCT assessment of bone formation around the defect-implant site. (A) X-ray image, 9µm voxels; (B) Vertical re-alignment of the implant in μCT dataset. (C) Selected ROI (white) based on implant landmarks.
