# Supplementary material for: The efficacy of a novel porcine-derived collagen membrane on guided bone regeneration: a comparative study in canine model
Source: BMC Oral Health. 2025 May 29;25:850. doi: 10.1186/s12903-025-05930-6 (PMC12123806; doi:10.1186/s12903-025-05930-6)
Supplement: Supplementary file 3 — Supplementary Material 3 [file 12903_2025_5930_MOESM3_ESM.docx]

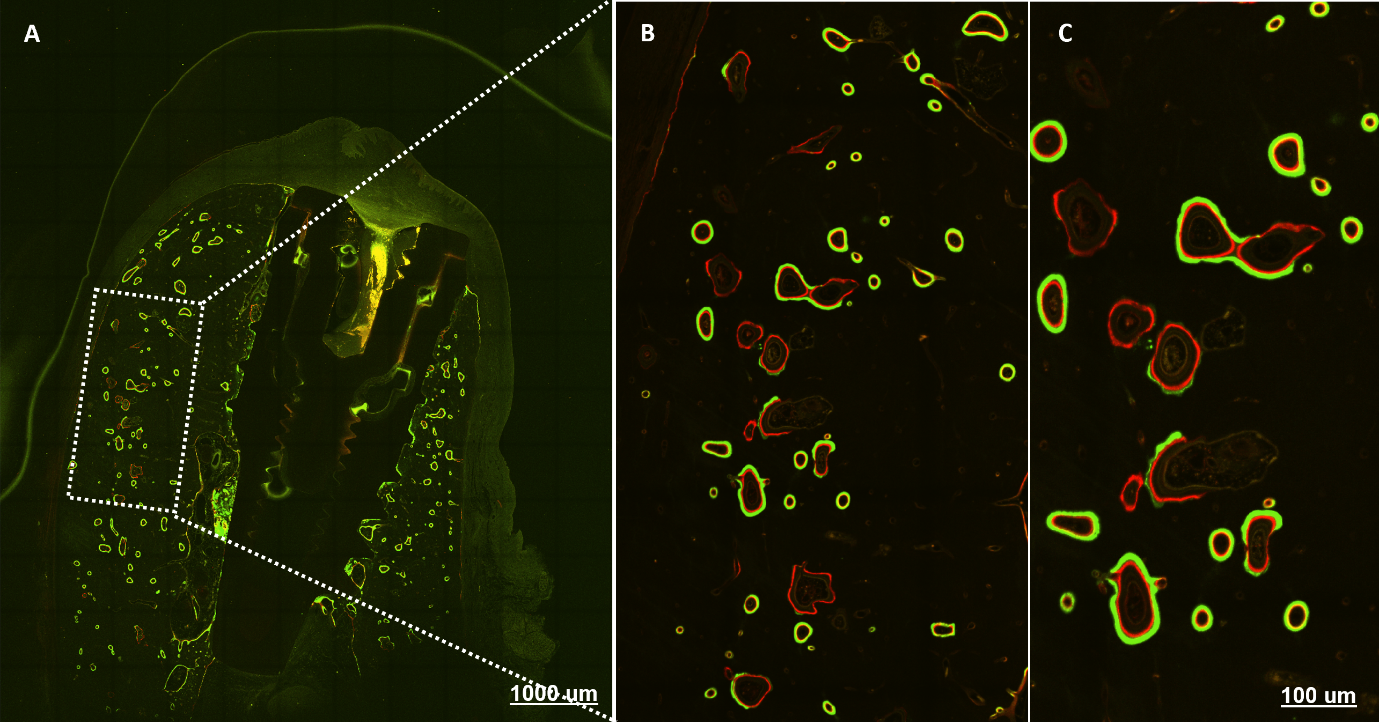


Supplementary Figure 4*.* Representative image of in vivo alizarin/calcein double-labelling for assessment of bone formation 12 weeks post-treatment. (A) Low magnification imaging of bone formation after alizarin/calcein double-labeling. Fluorochromes were intravenously injected 7 days apart and incorporated at sites of active bone mineralization. (B and C) High power magnification of bone formation sites. Alizarin complexone was used to label initial bone formation and calcein was used to label subsequent new bone formation 7 days later. The distance between the red and green fluorescence represents the amount of new bone formed during the 7-day period. Active bone mineralization was observed at 12 weeks in all treatment groups.
