# Supplementary material for: The efficacy of a novel porcine-derived collagen membrane on guided bone regeneration: a comparative study in canine model
Source: BMC Oral Health. 2025 May 29;25:850. doi: 10.1186/s12903-025-05930-6 (PMC12123806; doi:10.1186/s12903-025-05930-6)
Supplement: Supplementary file 4 — Supplementary Material 4 [file 12903_2025_5930_MOESM4_ESM.docx]

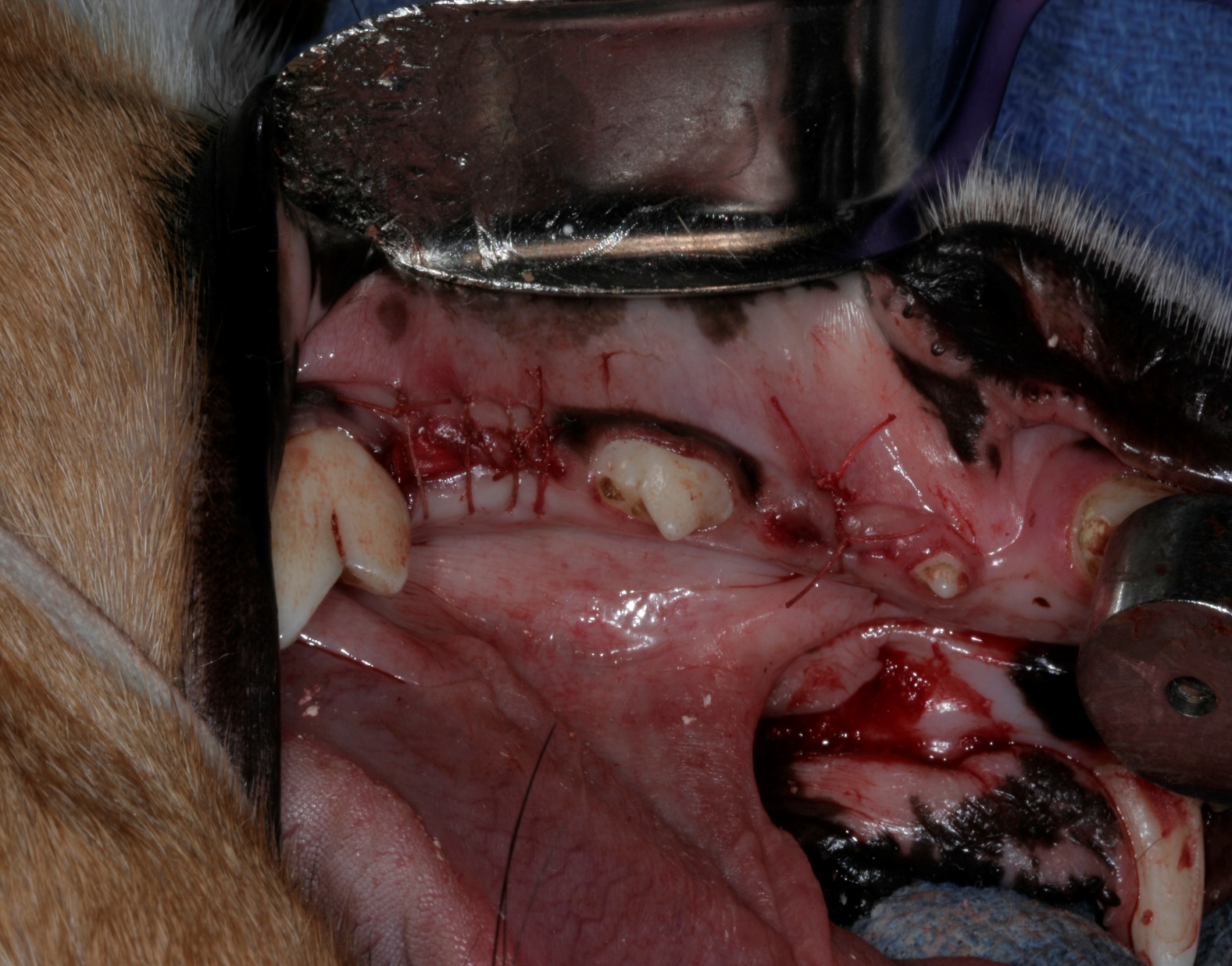

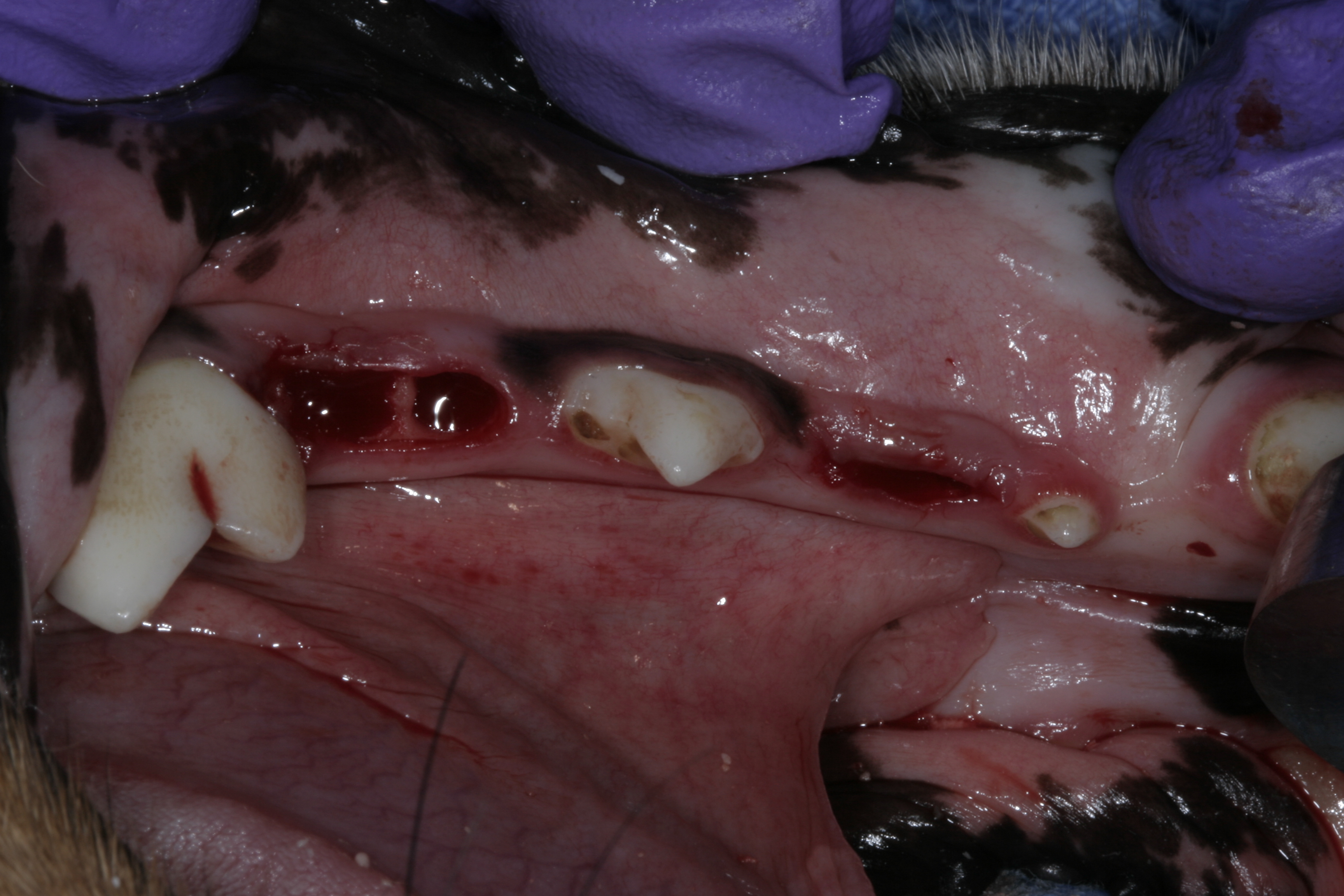

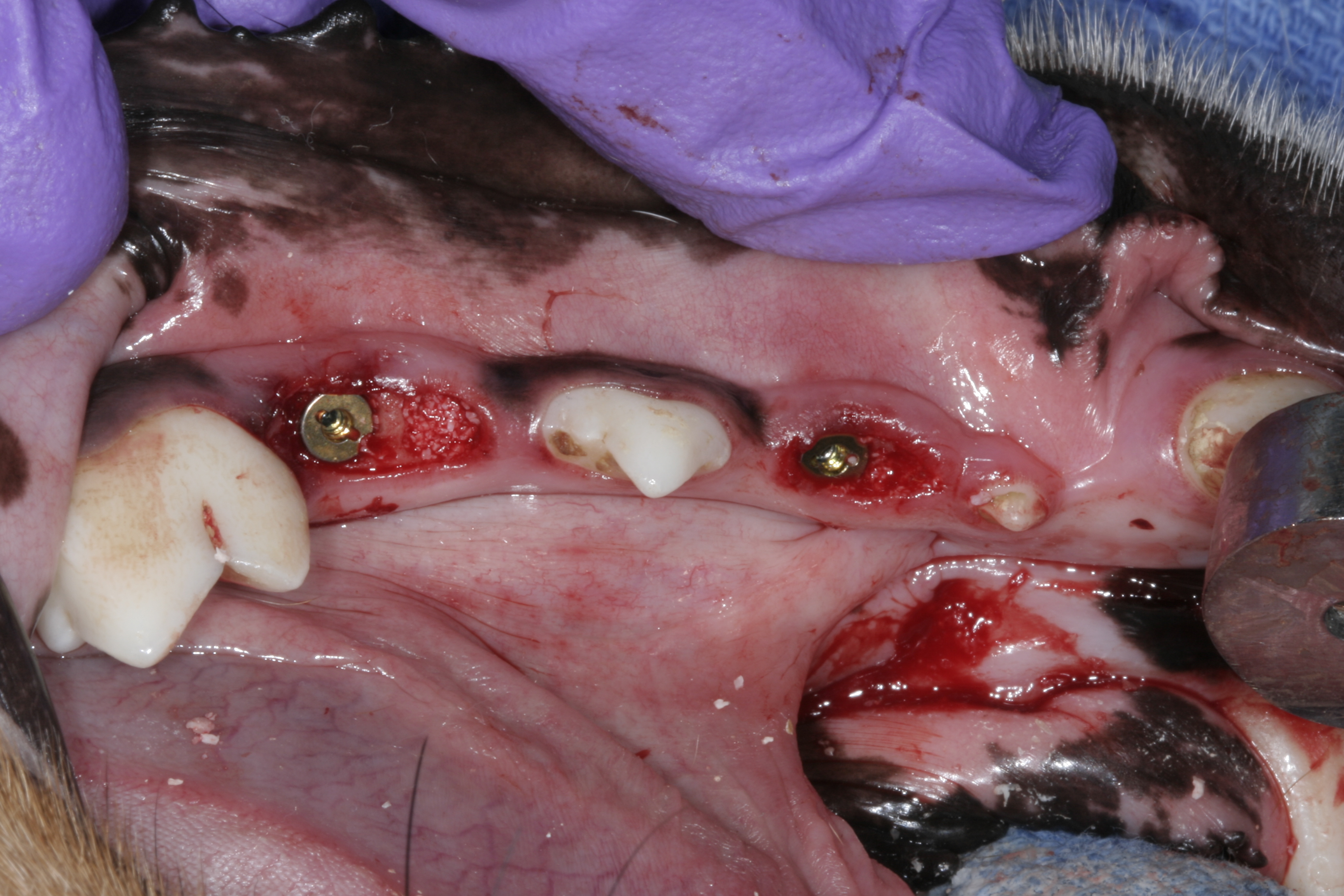

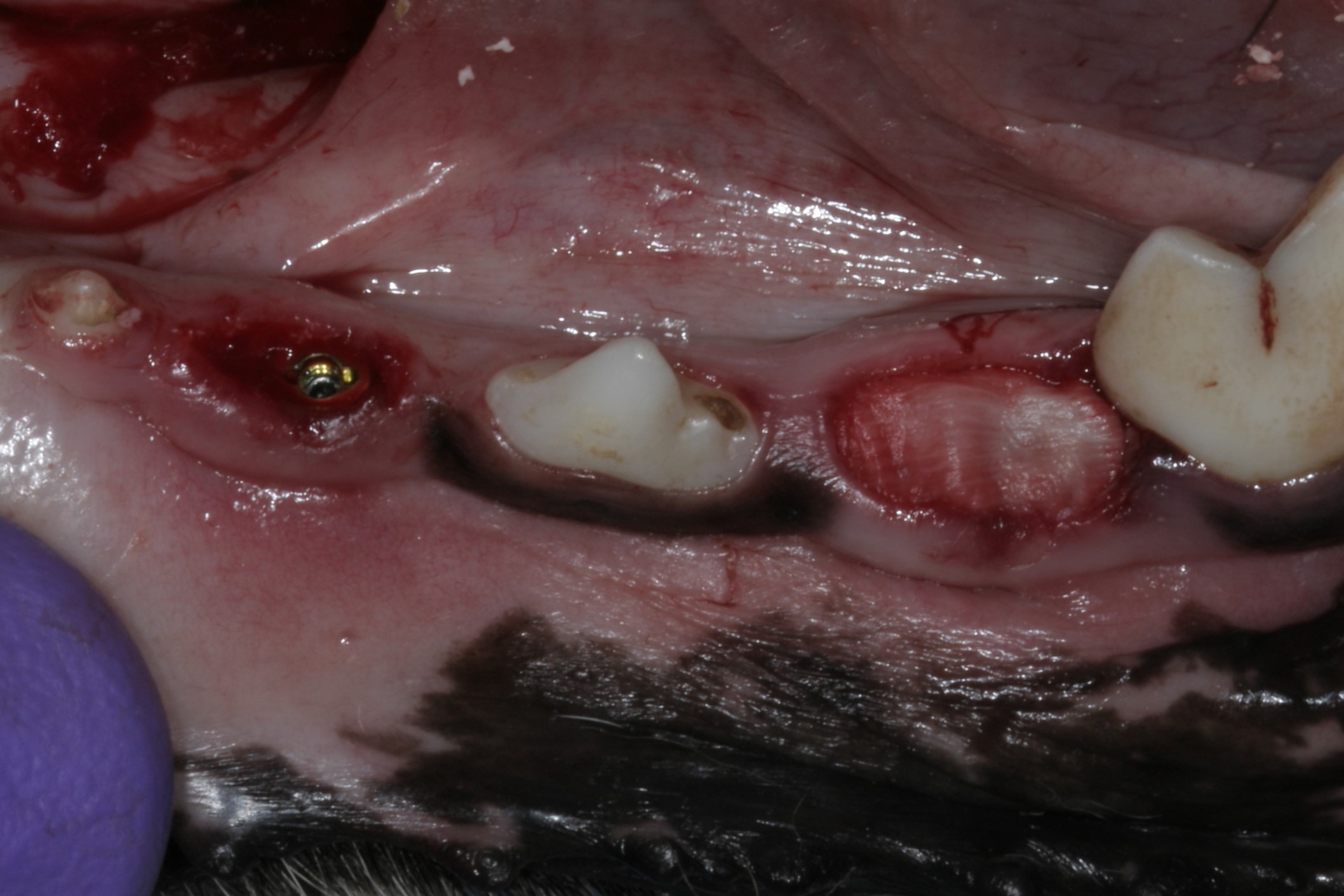


**A**

**D**

**C**

**B**

**2**

**1**

**Supplementary Figure 1*.*** Representative image of the surgical procedures on canine subjects. (A) Selected premolars were sectioned to facilitate atraumatic extraction. (B) A titanium threaded implant was then placed in the tooth socket and the remaining void was filled with Bio-Oss spongious bone substitute. (C) *Site 1:* No membrane was placed over the defect in the control site. *Site 2*: A collagen barrier membrane, either Bio-Gide or Striate+ (Orthocell), was placed over the defect and tucked under the gingiva, sealing the socket. (D) The gingiva was then closed with interrupted sutures to cover the membrane.
