# Supplementary material for: The efficacy of a novel porcine-derived collagen membrane on guided bone regeneration: a comparative study in canine model
Source: BMC Oral Health. 2025 May 29;25:850. doi: 10.1186/s12903-025-05930-6 (PMC12123806; doi:10.1186/s12903-025-05930-6)
Supplement: Supplementary file 5 — Supplementary Material 5 [file 12903_2025_5930_MOESM5_ESM.docx]

| *Supplementary Table 1.* Semi-quantitative evaluation of membrane barrier function | | | |
| --- | --- | --- | --- |
| **Epithelial ingrowth** | | **Membrane Resorption** | |
| **Observation** | **Score** | **Observation** | **Score** |
| No epithelial presence in bone defect | 0 | No resorption, membrane intact | 0 |
| Minimal epithelial presence in bone defect, no effect on bone regeneration | 1 | Minimal resorption, membrane body generally intact, some minor fragments around edges &/or minor discontinuities | 1 |
| Moderate epithelial presence in bone defect, bone regeneration may be affected | 2 | Moderate resorption, membrane body fragmented with some discontinuities between pieces | 2 |
| Marked epithelial presence in bone defect, bone regeneration affected | 3 | Marked resorption, membrane appears as widely distributed small fragments | 3 |
| Abundant epithelial cells in bone defect, minimal or no bone regeneration | 4 | No membrane fragments visible, appears to be completely resorbed | 4 |
